# Real-World Utilization of Palbociclib as First-Line Treatment for Canadian HR+/HER2− Women with Metastatic Breast Cancer: Results from PALCAN Study

**DOI:** 10.3390/curroncol33020081

**Published:** 2026-01-30

**Authors:** Daniel Rayson, Jonathan Bertin, Maxim Lemelin, Madeline Tong, Ryan Ng, Philip Ding, Winson Y. Cheung, Arushi Sharma, Phu Vinh On, Guillaume Feugère, Sasha Lupichuk

**Affiliations:** 1 Division of Medical Oncology, Dalhousie University, Halifax, NS B3H 2Y9, Canada; 2 Pfizer Canada ULC, Kirkland, QC H9J 2M5, Canadamaxim.lemelin@pfizer.com (M.L.); phuvinh.on@pfizer.com (P.V.O.); guillaume.feugere@pfizer.com (G.F.); 3 IQVIA Solutions Canada, Kirkland, QC H9J 2M5, Canada; madeline.tong@iqvia.com (M.T.); ryan.ng@iqvia.com (R.N.); arushi.fraelic@iqvia.com (A.S.); 4 Oncology Outcomes Research Program, University of Calgary, Calgary, AB T2N 4N1, Canada; philip.ding@oncoutcomes.com (P.D.); winson.cheung@oncoutcomes.com (W.Y.C.); sasha.lupichuk@albertahealthservices.ca (S.L.)

**Keywords:** Canada, HR+/HER2− breast cancer, palbociclib, real world, duration of treatment

## Abstract

Canadian real-world data (RWD) regarding palbociclib as a first-line therapy for patients with hormone receptor-positive (HR+), human epidermal growth factor receptor 2-negative (HER2−) metastatic breast cancer (MBC) is limited. The PALbociclib CANadian (PALCAN) study examined palbociclib utilization patterns as first-line treatment for HR+/HER2− MBC using Alberta health administrative data. The final PALCAN cohort included 472 female patients with a median age of 64 years and a median follow-up time of 22.8 months (IQR: 0.7–88.2). The median (95% CI) duration of treatment was 13.8 (12.7–15.1) months in the overall cohort (IQR: 5.6, 24.8 months), and the probability of treatment discontinuation within the first year was 45%. Aromatase inhibitors (AIs) and fulvestrant were the accompanying endocrine therapies (ETs) in 83% (N = 393) and 14% (N = 64) (15 with unknown accompanying therapy) of patients, respectively. The median duration of treatment for patients receiving an AI as an accompanying therapy was 15.1 (13.6–17.4) months and 7.9 months (5.8–12.6) for patients receiving fulvestrant, which may suggest endocrine resistance in the latter group. The PALCAN data provides insights into practice patterns and the effectiveness of palbociclib as a first-line therapy in female patients with HR+/HER2− breast cancer in the Canadian real-world setting.

## Simple Summary

There is limited Canadian real-world data that shows how palbociclib, a first-line treatment for hormone receptor-positive, human epidermal growth factor receptor 2-negative metastatic breast cancer, is being used in Canadian women. Using health administrative data from Alberta, Canada, this study examined the records of 472 female breast cancer patients who were inferred to have received palbociclib as first-line therapy and found that the median duration of treatment was 13.8 months and that the probability that these patients discontinued treatment within the first year was 45%. When patients receive palbociclib, they also receive an endocrine therapy in the form of an aromatase inhibitor (83% of patients in this study) or fulvestrant (14% of patients in this study). The median duration of treatment for patients receiving palbociclib with an aromatase inhibitor was 15.1 months, while the median duration of treatment for patients receiving palbociclib with fulvestrant was 7.9 months.

## 1. Introduction

Breast cancer is the most commonly diagnosed malignancy and the second leading cause of cancer-related deaths among Canadian women with an estimated 31,900 new cases and approximately 5400 deaths in 2025 [1]. Roughly 5–10% of patients are diagnosed with de novo metastatic disease, and 20–30% of women initially diagnosed with early-stage breast cancer will eventually develop metastases [2,3]. Five-year overall survival for metastatic breast cancer (MBC) among Canadian women is 23% compared to 89% for those with early-stage disease, reflecting a considerable unmet need in the advanced disease setting [4]. Breast cancer is categorized into molecular subtypes based on the expression of hormone receptors (HRs) (i.e., estrogen [ER] and progesterone [PR]) and human epidermal growth factor receptor 2 (HER2) [5]. The most prevalent subtype of breast cancer is HR+/HER2−, which accounts for about 70% of all breast cancer cases in Canada [6,7].

The combination of endocrine therapy with a cyclin-dependent kinase 4/6 (CDK4/6) inhibitor is a standard-of-care first-line treatment option for most patients with HR+/HER2− MBC [8,9,10]. Palbociclib was the first CDK4/6 inhibitor approved by Health Canada (2016) and is used in combination with aromatase inhibitors (AIs) or fulvestrant, with the latter commonly employed in cases of clinically suspected endocrine resistance [11]. Canadian approval was based on the PALOMA-2 phase III trial which demonstrated a significant improvement in progression-free survival (PFS) favoring palbociclib plus letrozole compared to single-agent letrozole in the first-line HR+/HER2− MBC setting (median PFS 24.8 vs. 14.5 months; *p* < 0.001) [12].

Patient heterogeneity is often underrepresented in clinical trials, and the importance of assessing the effectiveness of novel agents such as palbociclib in a diverse real-world patient population is increasingly recognized. Real-world data provide evidence on treatment effectiveness, which can complement data derived from randomized clinical trials (RCTs) and augment generalizability amongst a heterogeneous patient population more reflective of clinical practice. In 2021, a Canadian real-world study by Amaro et al. reported favorable population-based outcomes for CDK4/6 inhibitors in combination with AIs as first-line therapy for patients with HR+/HER2− MBC in Alberta [13]. More recently, a small single-institution study from Toronto, Ontario, examining the outcomes of 48 patients identified as having started a CDK4/6 inhibitor with endocrine therapy for HR+/HER2− MBC, observed a two-year overall survival (OS) rate of 97.4% with a median follow-up period of 28.7 months [14]. In both these cohort studies, palbociclib was the most commonly prescribed CDK4/6 inhibitor. Globally, multiple real-world and population-based studies have added to the data supporting the effectiveness of palbociclib combined with an aromatase inhibitor in routine clinical practice [15,16,17,18,19].

The aim of the PALbociclib CANadian (PALCAN) study was to examine population-based real-world data on palbociclib utilization patterns and the duration of treatment in the first-line treatment of Canadian patients with HR+/HER2− MBC.

## 2. Methods

### 2.1. Study Design and Setting

This was a population-based, retrospective cohort study involving the secondary use of de-identified health administrative data from Alberta, Canada.

#### Study Population and Cohort

This study included female patients aged 18 years and older with histologically confirmed HR+/HER2− MBC who received more than one cycle of palbociclib as first-line therapy for metastatic disease between 1 January 2016 and 30 September 2022 with a maximum follow-up until 31 December 2022 (end of study period). This means that patients had varying follow-up times from a potential minimum of 3 months to a maximum of 7 years, depending on the palbociclib initiation date. The index date was the date of the first prescription of palbociclib in combination with endocrine therapy. First-line palbociclib for MBC was defined as the administration of palbociclib after a breast cancer diagnosis and with no prior systemic therapy, including endocrine therapy. Endocrine therapy received more than 60 days prior to palbociclib initiation was considered adjuvant therapy, and these patients were included. Patients who received endocrine therapy 15–60 days before palbociclib initiation were considered as having received prior systemic therapy for MBC and therefore excluded. Patients who received endocrine therapy between 14 days prior and up to 28 days after palbociclib initiation were included. Patients starting palbociclib through an external access program prior to the public funding of palbociclib in 2018 may not be included in this study, which may account for the limited final patient counts. These study case definitions were required because information confirming the line of therapy was not available from the administrative data source described below.

### 2.2. Data Sources

The study cohort, including all cancer-related data and treatment information, was obtained from the Alberta Breast Data Mart (BDM), which collects information prospectively from the following: the Alberta Cancer Registry (ACR), which captures breast cancer diagnosis information; the Cancer Centre Electronic Medical Record (ARIA MO), which captures cancer treatment information; the Discharge Abstract Database (DAD), which captures data on hospitalizations; and the National Ambulatory Care Reporting System (NACRS), which captures information on emergency department (ED) visits. The BDM repository, which is now decommissioned, included all breast cancer patients residing in Alberta and diagnosed with early and metastatic breast cancer as of 1 January 2004 through October of 2022. The BDM captured information on demographics, certain tumor and clinical characteristics (e.g., histological confirmation for ER, PR, HER2 for de novo stage IV but not for relapsed stage IV subjected to repeat biopsy), surgical interventions, Cancer Care Alberta clinic visits, prescribed systemic therapies, vital status, and death/cause of death, if deceased. The ACR maintains data on all new cancer diagnoses and deaths in Alberta since 2004, and its quality, accuracy, and completeness are certified by the North American Association of Central Cancer Registries.

### 2.3. Outcomes, Variables, and Covariates

The primary study outcome was the estimation of the duration of treatment among patients receiving palbociclib in combination with endocrine therapy as first-line treatment for HR+/HER2− MBC. The duration of treatment was defined as the time (months) from the index date to the last date that palbociclib was prescribed plus the days’ supply of the last palbociclib prescription. Patients were censored at the earliest occurrence of death, end of the analysis period, or date of the last contact with Cancer Care Alberta/Alberta Health. The duration of treatment was analyzed across the entire cohort and by inferred menopausal status (defined as age < 50 vs. ≥50 years) and accompanying endocrine therapy (AI or fulvestrant). Menopausal status was not directly available within the dataset, and therefore an age cutoff was employed for stratification according to inferred menopausal status, consistent with other real-world data using administrative data with similar limitations [20].

Secondary outcomes included (i) the probability of palbociclib treatment discontinuation at one, two, and three years; (ii) treatment patterns (initial dose and duration of palbociclib; treatments received after palbociclib); (iii) time to next treatment (TTNT); and (iv) time to chemotherapy (TTC). TTNT was defined as the length of time (months) from the index date to the start date of a different systemic regimen. TTC was defined as the time (months) from the index date to the initiation of chemotherapy in any future line of treatment.

Available patient clinical characteristics and demographics were described, and a 1-year lookback period, prior to palbociclib initiation, was used to describe reported comorbidities utilizing the Charlson Comorbidity Index (CCI).

### 2.4. Statistical Analysis

The median duration of treatment was examined using Kaplan–Meier (KM) curves, with patients being censored at death, the end of the study period, or the date of the last contact, whichever occurred first. The probability of palbociclib discontinuation was derived from the KM curve at yearly intervals. Other time-to-event outcomes (TTNT and TTC) were reported by the Cumulative Incidence Function (CIF) using the Fine and Gray model to account for the competing risk of death. The 95% CI and *p*-values were calculated alongside the estimates, where applicable. Missing data were not imputed.

Descriptive statistics were reported as the mean and standard deviation (SD), median and interquartile range (IQR), minimum and maximum for continuous variables, and as frequency and percentage for categorical variables. Any results with small cell sizes from 1 to 10 were suppressed. Analysis was conducted using R Studio using R 4.3.1.

## 3. Results

**Figure 1:**
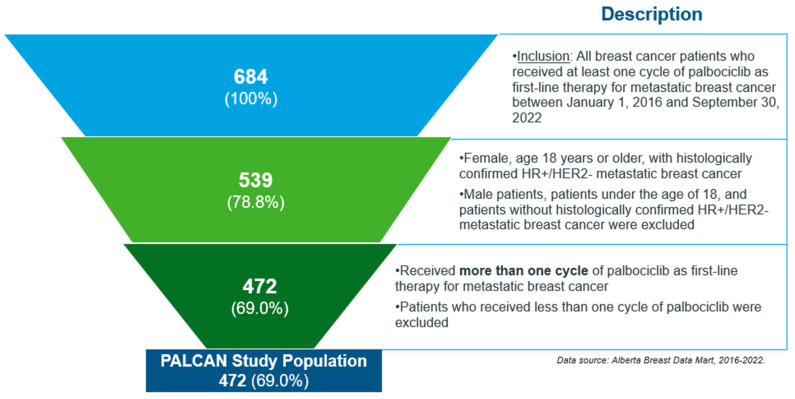
Patient flow diagram.

A total of 472 female patients aged 18 years or older with histologically confirmed HR+/HER2− MBC, who initiated treatment between 1 January 2016 and 30 September 2022, and who received more than one cycle of palbociclib as first-line therapy for MBC were included in the final study cohort (Figure 1).

### 3.1. Patient Demographics and Clinical Characteristics

The median age was 64 (IQR: 27–95) years, with approximately 87% of patients inferred as post-menopausal (age ≥ 50 years) (Table 1). The majority (86%) had disease that was positive for both estrogen (ER+) and progesterone receptors (PR+). De novo metastatic disease was reported in 45%, and 48% of these de novo patients had visceral involvement. A total of 33% of all patients had at least one comorbidity as defined by the CCI. As relapsed metastatic breast cancer is not reportable to the Alberta Cancer Registry, detailed biomarker and metastatic site and number data are limited to the de novo metastatic disease population. The median duration of follow-up was 22.8 (IQR: 0.7–88.2) months. Patient demographic and clinical characteristics stratified by inferred menopausal status are also available (Supplementary Table S1).

### 3.2. Treatment with Palbociclib and Accompanying Therapy

Within the PALCAN patient cohort, 90% initiated palbociclib at a dose level of 125 mg with 393 (86%) on concomitant AIs and 64 (14%) on fulvestrant (15 patients had missing information on the endocrine partner). Letrozole was the most commonly prescribed AI (n = 356; 91%). Amongst patients aged ≥50 and assumed to be post-menopausal, 85% received an AI and 13% fulvestrant in combination with palbociclib. Patients < 50 years of age had slightly lower AI usage, with 73% receiving an AI and 19% fulvestrant, both in combination with a luteinizing hormone-releasing hormone (LHRH) analog.

### 3.3. Duration of Treatment

**Figure 2:**
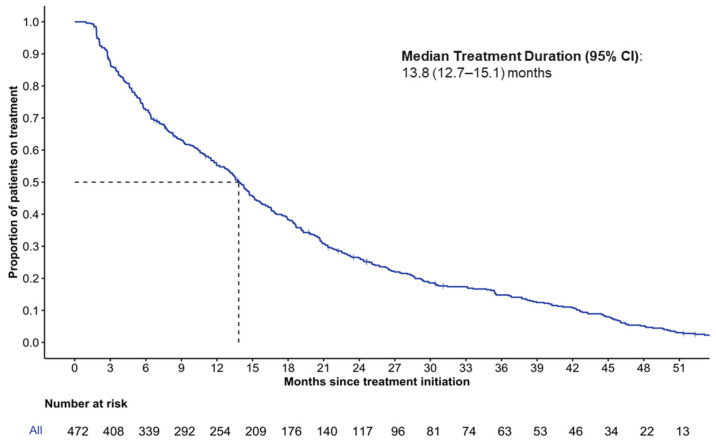
Kaplan–Meier estimation of first-line palbociclib duration of treatment.

The overall median (95% CI) duration of treatment for the entire cohort was 13.8 (12.7–15.1) months (IQR: 5.6, 24.8 months), with a median follow-up of 22.8 (95%CI: 0.7–88.2) months (Figure 2). The probability of treatment discontinuation (95% CI) at one, two, and three years following palbociclib initiation was 45% (40–49), 74% (69–77), and 85% (81–88), respectively, with the cumulative total number of patients censored at one, two, and three years being 25, 70, and 163 patients, respectively.

### 3.4. Duration of Treatment by Accompanying Therapy

**Figure 3:**
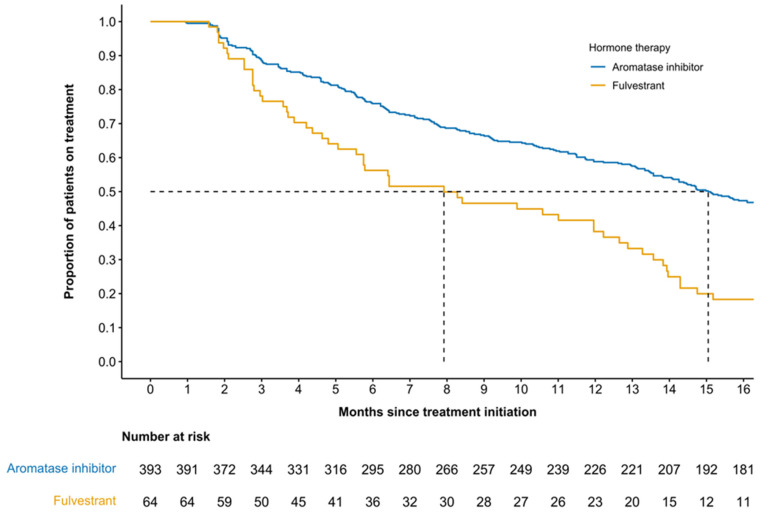
Kaplan–Meier estimation of first-line palbociclib duration of treatment by accompanying therapy.

The median (95% CI) duration of treatment was 15.1 (13.6–17.4) months for patients receiving palbociclib with an AI (Figure 3), with the probability of treatment discontinuation (95% CI) at one year being 41% (36–46), with a median (IQR) follow-up time of 26.3 (0.7–88.2) months.

Patients who received fulvestrant as accompanying therapy with first-line palbociclib had a median (95% CI) duration of treatment of 7.9 (5.8–12.6) months (Figure 3), with the probability of treatment discontinuation (95% CI) after one year being 62% (52–76). The median (IQR) follow-up time for this subgroup was 16.4 (1.2–35.0) months.

### 3.5. Duration of Treatment by Inferred Menopausal Status

In the assumed pre-menopausal subgroup (age < 50) with a median (IQR) follow-up time of 24.4 (2.1–72.4) months, the median (95% CI) duration of treatment was 16.8 (13.4–23.4) months, and the probability of treatment discontinuation (95% CI) at one year was 37% (24–48) (Supplemental Figure S1).

The median (95% CI) duration of treatment in the assumed post-menopausal subgroup (age ≥ 50) was 13.6 (12.0–14.8) months, with a probability of treatment discontinuation (95% CI) at one year of 46% (31–51) at a median (IQR) follow-up time of 22.8 (0.7–88.2) months (Supplemental Figure S1).

Additional study results on treatment patterns, including TTNT and TTC, can be found in the Supplementary Materials (Supplementary Figures S2 and S3; Supplementary Tables S2 and S3).

## 4. Discussion

The PALCAN study is a population-based cohort study examining real-world outcomes amongst a Canadian population of women receiving first-line therapy for HR+/HER2− MBC within the province of Alberta. To our knowledge, the PALCAN study represents the largest contemporaneous HR+/HER2− MBC Canadian patient cohort reported to date [3,13,14]. A total of 472 female patients were included, 90% of whom initiated palbociclib at the recommended dose of 125 mg. This is aligned with other real-world evidence such as the US Flatiron database study, a database study completed in Israel, and chart review studies completed in several countries (IRIS), all observing that more than 85% of patients initiated palbociclib at the standard starting dose [15,16,17,18,19].

The median duration of treatment in the overall patient cohort was 13.8 months, which is numerically lower than the 22.0 months reported in the PALOMA-2 trial, although a number of factors potentially account for this difference. This real-world patient population is more heterogenous than the trial patient population, which accrued patients based on strict inclusion and exclusion criteria [21]. Patient heterogeneity including comorbid conditions, inclusion of inferred pre-menopausal patients potentially presenting with more aggressive disease, socioeconomic status, family support systems, and access to care close to home may all impact a real-world patient population to a greater extent than a clinical trial population, potentially influencing treatment persistence and outcome. As this study was a retrospective health administrative database study, the definition of first-line treatment was inferred through a timing-based algorithm. The algorithm would have excluded patients who had started palbociclib through an external access program prior to public funding in 2018 who were still on treatment without progression. As the intent of treatment could not be clinically confirmed, the algorithm also may have included patients with endocrine-resistant disease receiving palbociclib with an AI beyond first-line. These scenarios would result in a lower median duration of therapy in the overall study cohort since patients who received palbociclib with fulvestrant had a median duration of therapy of only 7.9 months. As such, patients receiving palbociclib with an AI (median duration of treatment of 15.1 months) may be a more relevant metric to compare to clinical trial results such as those of PALOMA-2. The PALCAN study period ran between January 2016 and December 2022, which encompassed the full breadth of the COVID-19 pandemic, including the declaration of a provincial emergency in March 2020 which limited service access for oncology patients. This may have impacted treatment persistence due to the need for more frequent outpatient blood tests required with palbociclib versus single-agent endocrine therapy. A recent systematic review observed reductions in breast cancer screening and diagnoses, as well as higher proportions of patients presenting with later-stage cancer diagnoses during the COVID-19 pandemic [22]. A similar observation was made in this study, as de novo metastatic disease occurred in 45% of our cohort, which is higher than that reported in the Flatiron database (35% de novo disease, accrual between February 2016 and August 2018) and a previous study from Alberta (39% de novo disease, accrual between January 2016 and June 2019) [13,15].

As noted previously, in our cohort, 14% of patients received fulvestrant with palbociclib, and the median duration of treatment for those patients was significantly shorter than those receiving an AI. Among patients on palbociclib with an AI, the median duration of treatment was 15.1 months (95% CI: 13.6–17.4), which is close to that in two US-based real-world evidence studies leveraging electronic health record data from the Flatiron database and the US Oncology Network, which reported a median duration of treatment of 19.8 and 21.0 months, respectively [15,16]. Among patients who received palbociclib with fulvestrant, the median duration of treatment was 7.9 months (95% CI: 5.8–12.6) in the PALCAN study, which is comparable to a population-based retrospective cohort study in Portugal, where the median duration of treatment was 7.5 months for those receiving palbociclib with fulvestrant [23]. These observations suggest that fulvestrant was more often prescribed for those presumed to have endocrine-resistant disease (i.e., relapse less than 12 months after completion of adjuvant AI or during adjuvant therapy), with our results being comparable to those observed in the PALOMA-3 trial, where the median PFS in patients with endocrine-resistant disease receiving palbociclib plus fulvestrant was 7.4 versus 12.0 months for those with endocrine-sensitive disease [24].

In this study, we also stratified by inferred menopausal status. However, one of the limitations of using health administrative databases is the limited availability of clinical information such as menopausal status. Therefore, an age cutoff was employed to infer menopausal status, which is consistent with other real-world data studies using administrative databases that had similar constraints [20]. We found that patients assumed to be pre-menopausal had a median duration of treatment of 16.8 months, whereas patients assumed to be post-menopausal had a median duration of treatment of 13.6 months. Notably, only 62 patients were identified in the assumed pre-menopausal subgroup, compared to the 410 patients in the assumed post-menopausal subgroup. Because menopausal status could not be confirmed, we caution against comparing these values directly as this study was not designed for statistical comparisons between these two subgroups. However, through this study, we showed that in the Canadian real-world setting, there are patients who can be assumed to be pre-menopausal that are also receiving palbociclib with endocrine therapy as a first-line treatment for HR+/HER2− mBC and that the median duration of treatment of this subgroup was in line with the overall study population.

Study strengths include the utilization of Albertan health administrative data, which captures the entire provincial population of approximately 4.4 million people and includes all patients diagnosed with breast cancer within the province over the course of the study period, allowing for the assessment of longer-term real-world patient outcomes. Another strength is that the administrative data utilized was a subset of elements within a broad population-based health data system designed to examine population-based epidemiology and outcomes and may broadly represent the Canadian population. As this database was not intended to be used for research study-specific purposes, potential biases inherent in other types of real-world research such as recall, response, social acceptability, and sampling bias are minimized and likely eliminated.

Study limitations include the BDM having been decommissioned, so data are only limited to breast cancer patients diagnosed within Alberta from 2004 to October of 2022. This means that patients with early-stage disease prior to 2004 that recurred during our study period would not be captured, as well as those diagnosed beyond October 2022. In addition, patients who were diagnosed outside of Alberta but subsequently received treatment within the province were not included. Another limitation is the fact that health administrative data are not collected for research purposes and that there can be missing or erroneous data, which can limit and impact the data results. For example, the line of therapy could not be confirmed from the administrative data, so first-line palbociclib was derived based on the timing of other treatments received. Comorbidities and the CCI may be underreported because outpatient physician visits were not available and not all comorbidities may have been picked up during the one-year lookback period by ED visits and hospitalizations; for instance, well-managed chronic diseases are less likely to result in an ED visit or hospitalization and may not be reported if they did not affect patient care. An additional limitation to data interpretation is due to privacy legislation within Alberta, which stipulates that clinical or disease subsets with fewer than 10 patients cannot be reported and other categories must be suppressed to prevent back-calculation due to privacy concerns. PALCAN did not include chart abstraction and/or EMR review, which limits the description of certain clinical characteristics such as menopausal status (inferred based on age), potential endocrine resistance, metastatic presentation (visceral vs. non-visceral) for those with non-de novo metastatic breast cancer, response-related outcomes, and toxicities. Finally, the subgroup analyses were exploratory due to varying sample sizes, and only descriptive statistics were reported.

## 5. Conclusions

PALCAN assessed the real-world duration of treatment as a surrogate for the effectiveness of palbociclib as first-line therapy for female patients with HR+/HER2− breast cancer within the Canadian context, which furthers the understanding of palbociclib utility. Across real-world settings, variations in study design, cohort selection, patient characteristics, healthcare system, data access restrictions, and other contextual factors may contribute to differences in observed endpoints and outcomes such as the duration of treatment between health jurisdictions, as well as impacting study limitations. Overall, the PALCAN data builds upon existing evidence regarding the first-line use of palbociclib, aiding informed clinical decision-making and improving the understanding of the real-world clinical effectiveness of novel therapies.

## Figures and Tables

**Table 1:** Baseline clinical characteristics and demographics in the overall cohort and by the first-line hormone therapy agent (aromatase inhibitor and fulvestrant).

Characteristic	Overall, n (%) (n = 472)	Aromatase Inhibitor, n (%) (n = 393)	Fulvestrant n (%) (n = 64)
Age at index date, years			
Mean ± SD	64 ± 12	64 ± 12	66 ± 14
Median (IQR)	64 (27–95)	64 (27–90)	67 (36–95)
<50 years, n (%)	62 (13.1%)	45 (11.5%)	12 (18.8%)
50–70 years, n (%)	259 (54.9%)	228 (58.0%)	25 (39.1%)
>70 years, n (%)	151 (32.0%)	120 (30.5%)	27 (42.2%)
Inferred menopausal status, n (%)			
Assumed pre-menopausal (age < 50 years)	62 (13.1%)	45 (11.5%)	12 (18.8%)
Assumed post-menopausal (age ≥ 50 years)	410 (86.9%)	348 (88.5%)	52 (81.3%)
Hormone receptor status, n (%)			
ER+ only	59–67 *	43–51 *	4–12 *
PR+ only	<10 *	<10 *	<10 *
Both ER+ and PR+	404 (85.6%)	341 (86.8%)	51 (79.7%)
HER2 IHC score, n (%)			
0	57 (28.4%)	48 (29.4%)	<10 *
1	77 (38.3%)	63 (38.7%)	11 (39.3%)
2+ (ISH/FISH-negative)	67 (33.3%)	52 (31.9%)	13 (46.4%)
Unknown	271	230	31–39 *
Histopathology, n (%)			
Ductal	369 (78.2%)	307 (78.1%)	49 (76.6%)
Lobular	48 (10.2%)	1–47 *	<10 *
Other	55 (11.7%)	1–54 *	<10 *
Metastatic presentation, n (%)			
De novo	214 (45.3%)	186 (47.3%)	28 (28.1%)
Prior early breast cancer	258 (54.7%)	207 (52.7%)	46 (71.9%)
Metastatic disease distribution, n (%)			
Non-visceral	112 (52.3%)	95–103 *	9–17 *
Visceral	102 (47.7%)	91–101 *	<10 *
Unknown **	258	207	46
Number of metastatic sites, n (%)			
Unknown **	258 (54.7%)	207 (52.7%)	46 (71.9%)
1	100 (21.2%)	83–91 *	9–17 *
2+	114 (24.2%)	105–113 *	<10 *
Charlson comorbidity index, n (%)			
0	316 (66.9%)	266 (67.7%)	42 (65.6%)
1	116 (24.6%)	99 (25.3%)	12 (18.8%)
2+ or missing	40 (8.5%)	28 (7.0%)	10 (15.6%)
Vascular/cardiac ***, n (%)	29 (6.1%)	20 (5.1%)	<10 *
Gastrointestinal ***, n (%)	23 (4.9%)	15 (3.8%)	<10 *
Musculoskeletal ***, n (%)	13 (2.8%)	11 (2.8%)	<10 *
Metabolic ***, n (%)	48 (10.2%)	39 (9.9%)	<10 *
Median follow-up time, months (range)	22.8 (0.7–88.2)	26.3 (0.7–88.2)	16.4 (1.2–35.0)
Year of palbociclib initiation			
2016–2017	38 (8.1%)	29–37 *	<10 *
2018	103 (21.8%)	94–101 *	<10 *
2019	93 (19.7%)	84–92 *	<10 *
2020	84 (17.8%)	65 (16.5%)	18 (28.1%)
2021	103 (21.8%)	71 (18.1%)	27 (42.2%)
2022	51 (10.8%)	40 (10.2%)	11 (17.2%)

ER: Estrogen receptor; HER2: Human epidermal growth factor receptor 2; IHC: Immunohistochemistry; IQR: Interquartile range; PR: Progesterone receptor; SD: Standard deviation. For 15 patients included in the overall cohort, information on the accompanying endocrine therapy was missing. Visceral metastasis includes the adrenal gland, brain, liver, lung, pleura, and peritoneum. * Due to privacy legislation within Alberta, clinical or disease subsets with fewer than 10 patients cannot be reported, and other categories must be suppressed to prevent back-calculation due to privacy concerns. The * symbol indicates either small cells with fewer than 10 patients (reported as <10 *) or additional suppression required (reported as a range of patient values that would correspond to the 1 to 9 patients suppressed). ** Unknown was due to missing data for those with non-de novo metastatic disease. *** Comorbidities were defined as follows: “vascular/cardiac” = myocardial infarction, congestive heart failure, peripheral vascular disease, cerebrovascular disease; “gastrointestinal” = peptic ulcer disease, mild/moderate liver disease; “musculoskeletal” = rheumatic disease; “metabolic” = diabetes.

## Data Availability

Pfizer can provide access to related study documents (e.g., protocol, Statistical Analysis Plan [SAP]) upon request from qualified researchers and subject to certain criteria, conditions, and exceptions. The patient-level data are not publicly available due to privacy or ethical considerations. Further details on Pfizer’s data sharing criteria and process for requesting access can be found at https://www.pfizer.com/science/clinical-trials/trial-data-and-results/data-requests.

## Supplementary Materials

The following supporting information can be downloaded at https://www.mdpi.com/article/10.3390/curroncol33020081/s1, Figure S1: Kaplan–Meier estimation of first-line palbociclib duration of treatment by inferred menopausal status; Figure S2: Cumulative Incidence Function of time to next treatment with death as competing risk; Figure S3: Cumulative Incidence Function of time to chemotherapy with death as competing risk; Table S1: Descriptive statistics of baseline demographic and clinical characteristics, by inferred menopausal status; Table S2: Descriptive statistics of baseline demographic and clinical characteristics, by lines of treatment received; Table S3: Descriptive statistics of treatment patterns and treatment characteristics. References [25,26] are cited in the Supplementary Materials.
