# Supplementary material for: Real-World Utilization of Palbociclib as First-Line Treatment for Canadian HR+/HER2− Women with Metastatic Breast Cancer: Results from PALCAN Study
Source: Curr Oncol. 2026 Jan 30;33(2):81. doi: 10.3390/curroncol33020081 (PMC12938961; doi:10.3390/curroncol33020081)
Supplement: Supplementary file 1 [file curroncol-33-00081-s001.zip › curroncol-3963839-supplementary.pdf]

# Appendix

**List of Supplementary Figures:**


- **Supplemental Figure S1.** Kaplan-Meier estimation of first-line palbociclib duration of treatment by inferred menopausal status
- **Supplementary Figure S2.** Cumulative Incidence Function of Time to Next Treatment with death as a competing risk
- **Supplemental Figure S3.** Cumulative Incidence Function of Time to Chemotherapy with death as a competing risk


**List of Supplementary Tables:**


- **Supplementary Table S1.** Descriptive statistics of baseline demographic and clinical characteristics, by inferred menopausal status
- **Supplementary Table S2.** Descriptive statistics of baseline demographic and clinical characteristics, by lines of treatment received
- **Supplementary Table S3.** Descriptive statistics of treatment patterns and treatment characteristics

**Supplemental Figure S1. Kaplan-Meier estimation of first-line palbociclib duration of treatment by assumed menopausal status***

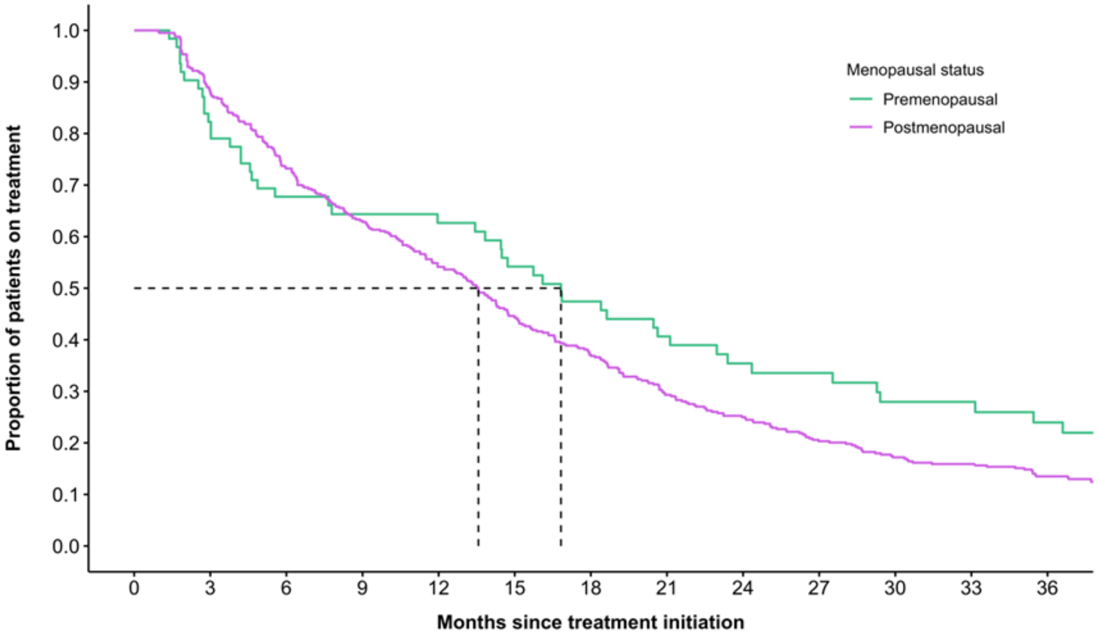


**Number at risk**

| | | | | | | | | | | | | | |
|---|---|---|---|---|---|---|---|---|---|---|---|---|---|
| Premenopausal | 62 | 51 | 42 | 38 | 37 | 32 | 28 | 24 | 19 | 18 | 15 | 14 | 12 |
| Postmenopausal | 410 | 357 | 297 | 254 | 217 | 177 | 148 | 116 | 98 | 78 | 66 | 60 | 51 |

*Age <50 vs ≥50 years was used as a determining factor for menopausal status.

In the assumed pre-menopausal subgroup (age < 50) with a median (IQR) follow-up time of 24.4 (2.1–72.4) months, the median (95% CI) duration of treatment was 16.8 (13.4–23.4) months and the probability of treatment discontinuation (95% CI) at one year was 37% (24–48). (Supplemental Figure 1).

Median (95% CI) duration of treatment in the assumed post-menopausal subgroup (age ≥ 50) was 13.6 (12.0–14.8) months, with a probability of treatment discontinuation (95% CI) at one year of 46% (31–51) at a median (IQR) follow-up time of 22.8 (0.7–88.2) months (Supplemental Figure 1).

**Supplemental Figure S2. Cumulative Incidence Function of Time to Next Treatment with death as a competing risk**

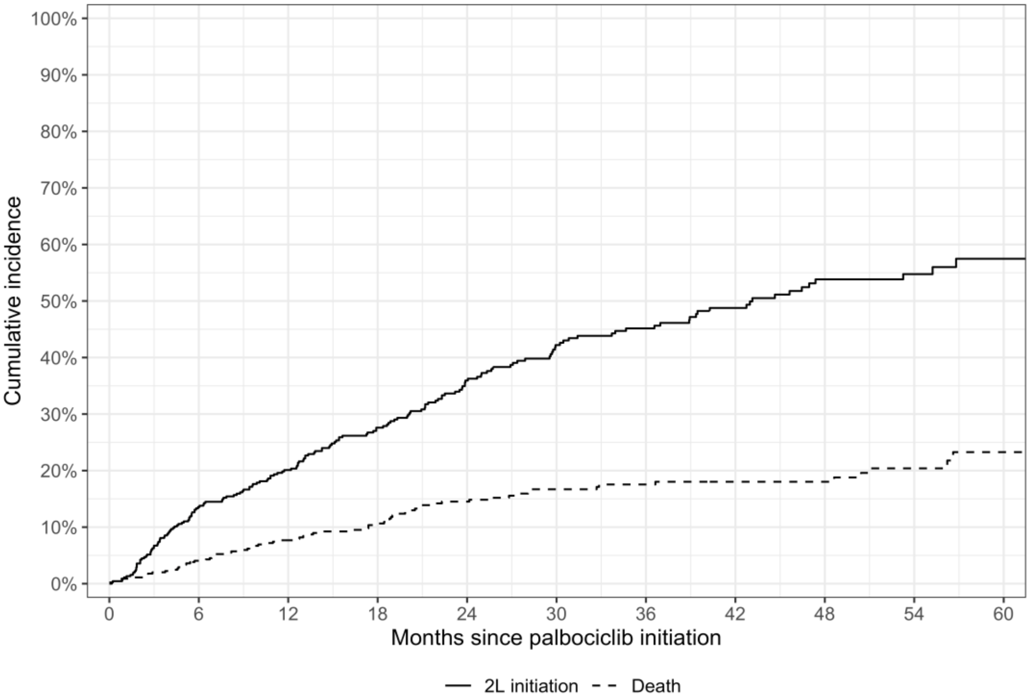


| | | | | | | | | | | | |
|---|---|---|---|---|---|---|---|---|---|---|---|
| At Risk | 452 | 353 | 286 | 214 | 150 | 102 | 78 | 59 | 37 | 23 | 10 |
| Events | 1 | 78 | 120 | 159 | 199 | 222 | 231 | 239 | 247 | 251 | 255 |

The probability of patients switching to second-line treatment after palbociclib initiation was 20% (95% CI: 16–24) at 1 year and 36% (95% CI: 31–41) at 2 years. The time to the median cumulative incidence is 43.1 months (95% CI: 34.1, 56.8).

**Supplemental Figure S3. Cumulative Incidence Function of Time to Chemotherapy with death as a competing risk**

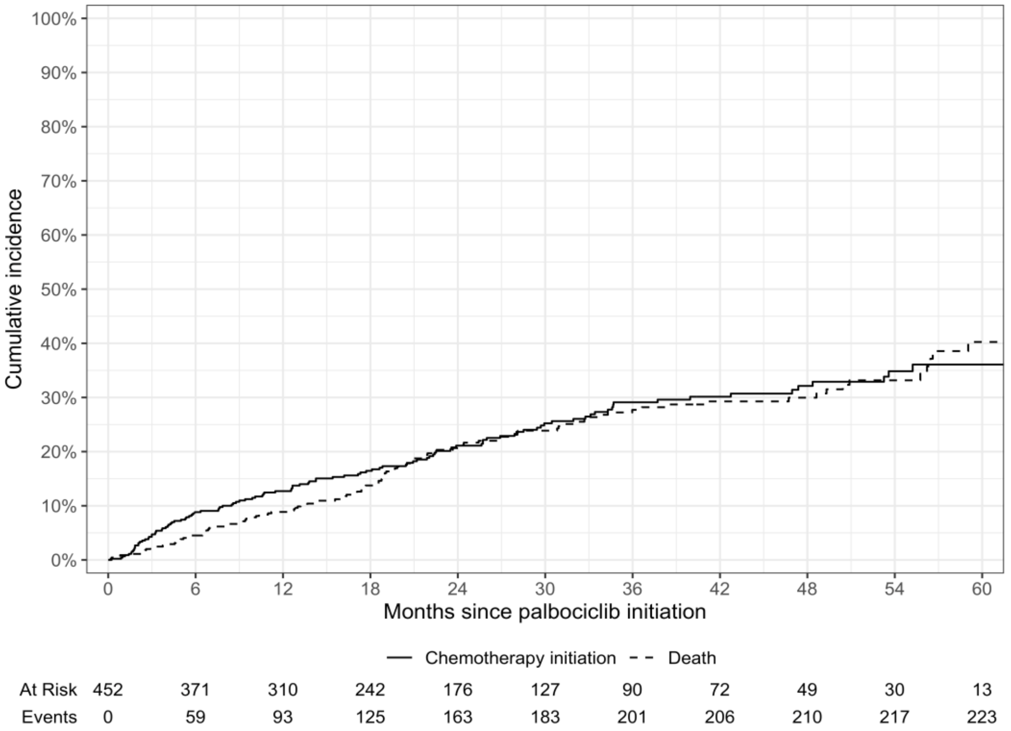


The probability of receiving chemotherapy in any line after palbociclib initiation was 13% (95% CI: 9.8–16.0) at 1 year and 21% (95% CI: 17–25) at 2 years, respectively.

*Supplemental Tables*

**Supplementary Table S1. Descriptive statistics of baseline demographic and clinical characteristics, by inferred menopausal status**

| Characteristic | Assumed Pre-menopausal Status (age < 50 years), n = 62 | Assumed Post-menopausal status (age ≥ 50 years), n = 410 |
|---|---|---|
| **Age at index date, years** | | |
| Mean ± SD | 43 ± 6 | 67 ± 10 |
| Median (Range) | 44 (27–50) | 67 (50–95) |
| **Hormone receptor status, n (%)** | | |
| ER+ | <10* | 53-61* |
| PR+ | <10* | <10* |
| Both ER+ and PR+ | 56 (90.3%) | 348 (84.9%) |
| **Disease morphology, n (%)** | | |
| Ductal | 50 (80.6%) | 319 (77.8%) |
| Lobular | <10* | 1-48* |
| Other | <10* | 1-55* |
| **Metastatic presentation, n (%)** | | |
| *De novo* | 22 (35.4%) | 192 (46.8%) |
| Prior early breast cancer | 40 (64.5%) | 218 (53.2%) |
| **Metastatic disease distribution, n (%)** | | |
| Non-visceral | 12 (54.5%) | 100 (52.1%) |
| Visceral | 10 (45.5%) | 92 (47.9%) |
| Unknown* * | 40 | 218 |
| **Number of metastatic sites, n (%)** | | |
| Unknown** | 40 (64.5%) | 218 (53.2%) |
| 1 | 11 (17.7%) | 89 (21.7%) |

| Characteristic | Assumed Pre-menopausal Status (age < 50 years), n = 62 | Assumed Post-menopausal status (age ≥ 50 years), n = 410 |
|---|---|---|
| 2+ | 11 (17.7%) | 103 (25.1%) |
| **HER2 IHC score, n (%)** | | |
| 0 | <10* | 1-56* |
| 1+ | 10 (52.6%) | 67 (36.8%) |
| 2+ (ISH/FISH negative) | <10* | 1-76* |
| Unknown | 43 | 228 |
| **Charlson Comorbidity Index, n (%)** | | |
| 0 | 48 (78.7%) | 268 (65.8%) |
| 1 | <10* | 1-115* |
| 2+ or missing | <10* | 1-39* |
| **Prior surgery, n (%)** | 46 (75.4%) | 248 (60.5%) |
| Missing | 1 | 0 |

ER: Estrogen receptor; HER2: Human epidermal growth factor receptor 2; IHC: Immunohistochemistry; IQR: Interquartile range; PR: Progesterone receptor; SD: Standard deviation.

To minimize patient re-identification, cells with 1-9 patients were suppressed and reported as <10.

* indicates further data suppression to prevent back-calculation of cells with 1-9 and patients reported as <10. Cells with * were reported as a range that correspond to the suppression from the <10 cells.

** Unknown was due to missing data for those with non-*de novo* metastatic disease.

**Supplementary Table S2. Descriptive statistics of baseline demographic and clinical characteristics, by lines of treatment received**

| | Lines of treatment received | | |
|---|---|---|---|
| **Characteristic** | **1, n = 288** | **2, n = 115** | **≥3, n = 69** |
| **Age at index date, years** | | | |
| Mean ± SD | 66 ± 12 | 63 ± 13 | 58 ± 11 |
| Median (IQR) | 67 (32–95) | 62 (33–88) | 57 (27–82) |
| <50 years, n (%) | 27 (9.4%) | 20 (17.4%) | 15 (21.7%) |
| 50-70 years, n (%) | 154 (53.5%) | 59 (51.3%) | 46 (66.7%) |
| >70 years, n (%) | 107 (37.2%) | 97-107* | <10* |
| **Menopausal status, n (%)** | | | |
| Assumed pre-menopausal (age <50 years) | 27 (9.4%) | 20 (17.4%) | 15 (21.7%) |
| Assumed post-menopausal (age ≥50 years) | 261 (90.6%) | 95 (82.6%) | 54 (78.3%) |
| **Hormone receptor status, n (%)** | | | |
| ER+ | 33-41* | 10-18* | <10* |
| PR+ | <10* | <10* | <10* |
| Both ER+ and PR+ | 246 (85.4%) | 96 (83.5%) | 62 (89.9%) |
| **Disease morphology, n (%)** | | | |
| Ductal | 224 (77.8%) | 97 (84.3%) | 48 (69.6%) |
| Lobular | 29 (10.1%) | <10* | 10-18* |
| Other | 35 (12.2%) | <10* | 11-19* |
| **Metastatic presentation, n (%)** | | | |
| *De novo* | 136 (47.2%) | 44 (38.3%) | 34 (49.3%) |
| Early breast cancer | 152 (52.8%) | 71 (61.7%) | 35 (50.7%) |
| **Metastatic disease distribution, n (%)** | | | |
| Non-visceral | 65 (47.8%) | 29 (65.9%) | 18 (52.9%) |

| | Lines of treatment received | | |
|---|---|---|---|
| **Characteristic** | **1, n = 288** | **2, n = 115** | **≥3, n = 69** |
| Visceral | 71 (52.2%) | 15 (34.1%) | 16 (47.1%) |
| Unknown** | 152 | 71 | 35 |
| **Number of metastatic sites, n (%)** | | | |
| Unknown** | 152 (52.8%) | 71 (61.7%) | 35 (50.7%) |
| 1 | 55 (19.1%) | 27 (23.5%) | 18 (26.1%) |
| 2+ | 81 (28.1%) | 17 (14.8%) | 16 (23.2%) |
| **HER2 IHC score, n (%)** | | | |
| 0 | 41 (30.4%) | 7-15* | <10* |
| 1+ | 53 (39.3%) | 15-23* | <10* |
| 2+ (ISH/FISH negative) | 41 (30.4%) | 17-25* | <10* |
| Unknown | 153 | 69 | 49 |
| **Charlson Comorbidity Index, n (%)** | | | |
| 0 | 187 (65.6%) | 76 (66.7%) | 53 (76.8%) |
| 1 | 75 (26.3%) | 30-38* | 7-15* |
| 2+ or unknown | 26 (9.0%) | <10* | <10* |
| **Vascular/cardiac comorbidity***, n (%)** | 1-11* | <10* | <10* |
| **Gastrointestinal comorbidity***, n (%)** | 11 (3.8%) | <10* | <10* |
| **Musculoskeletal comorbidity***, n (%)** | <10* | <10* | <10* |
| **Metabolic comorbidity***, n (%)** | 32 (11.1%) | 7-15* | <10* |
| **Prior surgery, n (%)** | 177 (61.5%) | 76 (66.1%) | 42 (60.3%) |

ER: Estrogen receptor; HER2: Human epidermal growth factor receptor 2; IHC: Immunohistochemistry; IQR: Interquartile range; PR: Progesterone receptor; SD: Standard deviation.

*Due to privacy legislation within Alberta, clinical or disease subsets with fewer than 10 patients cannot be reported and other categories must be suppressed to prevent back calculation due to privacy concerns. The (*) indicates either: small cells with fewer than 10 patients (reported as <10*) or additional suppression required (reported as a range of patient values that would correspond to the 1 to 9 patients suppressed).

** Unknown was due to missing data for those with non-*de novo* metastatic disease.

*** Co-morbidities were defined as follows: "vascular/cardiac" = myocardial infarction, congestive heart failure, peripheral vascular disease, cerebrovascular disease; "gastrointestinal" = peptic ulcer disease, mild/moderate liver disease; "musculoskeletal" = rheumatic disease; "metabolic" = diabetes.

**Supplementary Table S3. Descriptive statistics of treatment patterns and treatment characteristics**

| Characteristic | N | n (%) |
|---|---|---|
| **Lines of therapy received** | 472 | |
| First-line | | 472 (100.0%) |
| Second-line | | 184 (39.0%) |
| Third-line or more | | 69 (14.6%) |
| **Initial palbociclib dose, mg** | 354 | |
| 75 | | <10* |
| 100 | | 27-35* |
| 125 | | 318 (89.8%) |
| Missing | | 118 |
| **First-line hormone therapy agent** | 457 | |
| Letrozole | | 356 (77.9%) |
| Fulvestrant | | 64 (14.0%) |
| Anastrozole | | 22 (4.8%) |
| Exemestane | | 15 (3.3%) |
| Missing | | 15 |
| **Second-line therapy type**** | 213 | |
| Chemotherapy | | 88 (41.3%) |
| Aromatase inhibitor | | 59 (27.7%) |
| Fulvestrant | | 22 (10.3%) |
| LHRH agonist | | 17 (8.0%) |
| Hormone therapy | | 12 (5.6%) |
| Targeted therapy | | 12 (5.6%) |
| Other | | 3 (1.4%) |

| Characteristic | N | n (%) |
|---|---|---|
| **Second-line therapy agent**** | 213 | |
| Capecitabine | | 70 (32.9%) |
| Exemestane | | 51 (23.9%) |
| Fulvestrant | | 22 (10.3%) |
| Leuprolide Acetate | | 16 (7.5%) |
| Tamoxifen Citrate | | 12 (5.6%) |
| Everolimus | | 10 (4.7%) |
| Other* | | 32 (15.0%) |
| **Third-line therapy type**** | 75 | |
| Chemotherapy | | 52 (69.3%) |
| Hormone therapy | | 10 (13.3%) |
| Other* | | 13 (17.3%) |
| **Third-line therapy agent**** | 75 | |
| Capecitabine | | 28 (37.3%) |
| Tamoxifen Citrate | | 10 (13.3%) |
| Other* | | 37 (49.3%) |
| **Received any chemotherapy** | 472 | |
| No; alive | | 248 (52.5%) |
| No; deceased | | 111 (23.5%) |
| **Therapy sequence (patients who received two lines)** | 184 | |
| Palbociclib, Letrozole \| Capecitabine | | 42 (22.8%) |
| Palbociclib, Letrozole \| Exemestane | | 39 (21.2%) |
| Palbociclib, Fulvestrant \| Capecitabine | | 16 (8.7%) |
| Palbociclib, Letrozole \| Fulvestrant | | 11 (6.0%) |
| Palbociclib, Letrozole \| Leuprolide Acetate | | 11 (6.0%) |
| Other[#] | | 65 (35.3%) |

| Characteristic | N | n (%) |
|---|---|---|
| **Therapy sequence (patients who received three lines)** | 69 | |
| Palbociclib, Letrozole \| Exemestane \| Capecitabine | | 10 (14.5%) |
| Other[#] | | 59 (85.5%) |

LHRH: Luteinizing hormone releasing hormone; SD: Standard deviation.

The percentages shown are based on eligible patients at each line.

[#]Second-line "other" agents include: anastrozole, doxorubicin, docetaxel, alpelisib, carboplatin, cyclophosphamide, ribociclib, fluorouracil, gemcitabine, goserelin acetate, letrozole, placebo/alpelisib. Third-line "other" agents include: doxorubicin, gemcitabine, cyclophosphamide, exemestane carboplatin, fulvestrant, letrozole, vinorelbine, docetaxel, anastrozole, everolimus, and leuprolide acetate.

*Due to privacy legislation within Alberta, clinical or disease subsets with fewer than 10 patients cannot be reported and other categories must be suppressed to prevent back calculation due to privacy concerns. The (*) indicates either: small cells with fewer than 10 patients (reported as <10*) or additional suppression required (reported as a range of patient values that would correspond to the 1 to 9 patients suppressed).

**Patients may have received more than one therapy for 2L and 3L therapy (combination therapy). As such, the therapies reported are not mutually exclusive, and total therapies used can sum to more than the total patients in 2L and 3L.

The majority of patients (86%) were given palbociclib in combination with AI (mainly letrozole), followed by combination with fulvestrant (14%). A real-world study conducted in Canada reported similar data – about 87% of patients received palbociclib plus letrozole and 13% received palbociclib plus fulvestrant (Mycock et al., 2021)[1]. The most common second-line therapy type after palbociclib was chemotherapy (41.3%), followed by AI (27.7%); the most common second-line agents were capecitabine (32.9%) and exemestane (23.9%). The most common sequence of treatments in patients who received second-line treatment was palbociclib and letrozole as first-line therapy, followed by capecitabine as second-line treatment in 22.8% of patients.

Among patients who received third-line treatment, chemotherapy was used by 69.3% of patients and the most common treatment was capecitabine (37.3%). The most common sequence of treatments in patients who received third-line treatment was palbociclib and letrozole as first-line therapy, followed by exemestane as second-line treatment and capecitabine as third-line treatment in 14.5% of patients.

Chemotherapy was the most common second- as well as third-line therapy, and capecitabine was the most frequently prescribed agent in both lines. The choice of chemotherapy as the next treatment option is aligned with the evidence that suggests that the recommended treatment for HR+/HER2− breast cancer as first-line therapy are CDK4/6 inhibitor plus aromatase inhibitor followed by endocrine therapy or chemotherapy depending on endocrine sensitivity/resistance (Schneeweiss et al. 2021)[2].

**Supplementary References**


1 Mycock, K., Zhan, L., Taylor-Stokes, G., Milligan, G. & Mitra, D. Real-World Palbociclib Use in HR+/HER2- Advanced Breast Cancer in Canada: The IRIS Study. *Curr Oncol* **28**, 678-688 (2021). https://doi.org:10.3390/curroncol28010066

2 Schneeweiss, A. *et al.* AGO Algorithms for the Treatment of Breast Cancer: Update 2021. *Geburtshilfe Frauenheilkd* **81**, 1101-1111 (2021). https://doi.org:10.1055/a-1519-7089

3 Kim, J.-Y., Shin, J., Ahn, J. S., Park, Y. H. & Im, Y.-H. Real World Experience of Second-Line Treatment Strategies after Palbociclib and Letrozole: Overall Survival in Metastatic Hormone Receptor-Positive Human Epidermal Growth Factor Receptor 2-Negative Breast Cancer. *Cancers* **15**, 3431 (2023).
